# Application of Bioinformatics Analysis to Identify Important Pathways and Hub Genes in Ovarian Cancer Affected by WT1

**DOI:** 10.3389/fbioe.2021.741051

**Published:** 2021-10-06

**Authors:** Kai Meng, Jinghe Cao, Yehao Dong, Mengchen Zhang, Chunfeng Ji, Xiaomei Wang

**Affiliations:** ^1^ Collaborative Innovation Center for Birth Defect Research and Transformation of Shandong Province, Jining Medical University, Jining, China; ^2^ Affiliated Hospital of Jining Medical University, Jining, China; ^3^ College of Basic Medicine, Jining Medical University, Jining, China

**Keywords:** Wilms tumor gene, ovarian cancer, RNA-sequencing, differentially expressed genes, bioinformatic analysis

## Abstract

Wilms tumor gene (WT1) is used as a marker for the diagnosis and prognosis of ovarian cancer. However, the molecular mechanisms involving WT1 in ovarian cancer require further study. Herein, we used bioinformatics and other methods to identify important pathways and hub genes in ovarian cancer affected by WT1. The results showed that WT1 is highly expressed in ovarian cancer and is closely related to the overall survival and progression-free survival (PFS) of ovarian cancer. In ovarian cancer cell line SKOV3, WT1 downregulation increased the mRNA expression of 638 genes and decreased the mRNA expression of 512 genes, which were enriched in the FoxO, AMPK, and the Hippo signaling pathways. The STRING online tool and Cytoscape software were used to construct a Protein-protein interaction (PPI) network and for Module analysis, and 18 differentially expressed genes (DEGs) were selected. Kaplan-Meier plotter analysis revealed that 16 of 18 genes were related to prognosis. Analysis of GEPIA datasets indicated that 7 of 16 genes were differentially expressed in ovarian cancer tissues and in normal tissues. The expression of IGFBP1 and FBN1 genes increased significantly after WT1 interference, while the expression of the SERPINA1 gene decreased significantly. The correlation between WT1 expression and that of these three genes was consistent with that of ovarian cancer tissues and normal tissues. According to the GeneMANIA online website analysis, there were complex interactions between WT1, IGFBP1, FBN1, SERPINA1, and 20 other genes. In conclusion, we have identified important signaling pathways involving WT1 that affect ovarian cancer, and distinguished three differentially expressed genes regulated by WT1 associated with the prognosis of ovarian cancer. Our findings provide evidence outlining mechanisms involving WT1 gene expression in ovarian cancer and provides a rational for novel treatment of ovarian cancer.

## Introduction

Ovarian cancer is the seventh most common cancer among women worldwide and the deadliest cancer among gynecology patients (Jemal et al., 2011; Siegel et al., 2015; Lheureux et al., 2019a). At present, there is no accurate early screening method for ovarian cancer, and most ovarian cancers are diagnosed in advanced stages (Lheureux et al., 2019b). The main treatment for ovarian cancer is surgical resection combined with chemotherapy. Despite its beneficial effects, long-term treatment will produce drug resistance to the selected target (Miller et al., 2009; Beaver et al., 2019). Moreover, the overall 5-years survival rate of ovarian cancer has not changed significantly since the 1980s, and the 5-years survival rate has remained constant at about 40% (Vaughan et al., 2011; Allemani et al., 2018). Thus, the mechanisms underlying occurrence and development of ovarian cancer need to be further clarified, and will provide important guidance for early diagnosis, improvement of prognosis, and more precise treatment of ovarian cancer.

The WT1 gene was cloned in 1990 (Gessler et al., 1990) and it was originally described as a classic tumor suppressor gene (Yang et al., 2007). However, WT1 is overexpressed in many hematological tumors and solid tumors, where it can also act as an oncogene (Loeb and Sukumar, 2002; Zhang et al., 2020). WT1 is an important therapeutic target, and a peptide vaccine has been developed for cancer treatment (Izumoto et al., 2008; Oka and Kawase, 2008). In addition, studies have shown that WT1 can influence the progression of cancer through a variety of genes or signaling pathways. For example, in the study of glioblastoma, WT1 may affect the viability and chemoresistance of glioblastoma cells *in vitro* by regulating the expression of the insulin-like growth factor 1 receptor (IGF1-R) Pathway (Chen et al., 2011). As far as breast cancer cells are concerned, WT1 may influence tumor cell growth by regulating the destabilization of β-catenin (Zhang et al., 2003). In lung cancer, WT1 may affect the oncogenesis of lung cancer through the PI3K/AKT signaling pathway (Wang et al., 2013). In addition, studies have reported that WT1 may affect the autophagy of osteosarcoma through AKT/JNK signaling (Mo et al., 2016).

Previous studies have shown that the WT1 gene is highly expressed in epithelial ovarian cancer tissues (Hylander et al., 2006), and can regulate the metastasis and invasion of ovarian cancer through the E-cadherin and ERK1/2 signaling pathways (Barbolina et al., 2008; Han et al., 2020). WT1 is also used as a marker for the diagnosis and prognosis of ovarian cancer (Taube et al., 2016; Carter et al., 2018; Mondal et al., 2021). However, the signaling pathways, functional pathways, and key genes involved in WT1 regulation of ovarian cancer need to be further defined.

Transcriptome technology can detect the expression of all genes at a specific time point, and is suitable for screening differentially expressed genes (DEGs). We used transcriptome technology and bioinformatics analysis to study the specific mechanisms induced by WT1 in regulating the process of ovarian cancer. In general, our systematic analysis will further elucidate the specific role played by WT1 in the pathogenesis of WT1 of ovarian cancer at the molecular level. Our findings will provide important guidance for the early diagnosis, improvement of prognosis, and precise treatment of ovarian cancer.

## Materials and Methods

### Cell Culture

The Human ovarian cancer cell line SKOV3 (Fenghui Biotechnology, Co.) was cultured in McCoy’s 5A medium, Coav-3 (Procell) and A2780 (BNCC) cells were cultured in DMEM, and OVCAR3 (BNCC) cells were cultured in RPMI-1640. Each medium was supplemented with 10% FBS at 37°C and 5% CO2. mRNA and protein were extracted when the cell lines reached 80% confluence. mRNA and protein extracts from the cells were stored at −80°C until use.

### Transfection of Wilms tumor gene1 Gene siRNA Sequence Into Ovarian Cancer Cell Line SKOV3

To study the function of WT1 in ovarian cancer cells, SKOV3 cells were cultured as previously described with minor modifications (Meng et al., 2019a; Meng et al., 2019b). Briefly, siRNA transfection was performed when the cells reached 50% confluence, and the culture medium was changed after 24 h and the culture continued for an additional 24 h siRNA was transfected into SKOV3 using Lipofectamine™ RNAiMAX according to the manufacturer’s instructions. The siRNA sequences of WT1 were 5′-GGA​CUG​UGA​ACG​AAG​GUU​UTT-3′ (forward) and 5′-AAA​CCU​UCG​UUC​ACA​GUC​CTT-3′ (reverse).

### RNA-Sequencing

Total RNA was processed using the mRNA enrichment method. Briefly, magnetic beads carrying OligodT were used to enrich mRNA with the polyA tail, which was then fragmented to obtain mRNA, and a single cDNA strand was synthesized, followed by synthesis of a double-stranded cDNA. After purification and recovery, sticky-end repair, the 3′-end with the “A" tail was ligated and the fragment size selection, PCR amplification was performed, the constructed library was checked for quality, and was then sequenced. Quality control (QC) and filtering were performed on the data obtained by sequencing. This project used the filtering software SOAPnuke independently developed by BGI for filtering RNA sequences. After obtaining clean reads, HISAT was used to align the clean reads to the reference genome sequence. After the comparison, a second QC for alignment of sequences was performed. After the second QC was passed, gene quantification and gene expression levels were determined. A total of 6 samples were tested using the DNBSEQ platform, and each sample produced an average of 6.58 GB of data. The average comparison rate of the sample comparison genome was 88.41%, and the average comparison rate of the comparison gene set was 74.87%. A total of 17,063 genes were detected.

### Identification of Differentially Expressed Genes

We set |log fold change (FC)|≧0.5, Q-value < 0.01 to identify DEGs between WT1 interference group (SKSi) and control group (SKNCmix). Sequencing results showed that the number of DEGs was 1,150. Compared with the control group, 638 genes in the WT1 interference group were significantly up-regulated and 512 genes were significantly down-regulated. We applied Dr. Tom’s online tool to draw volcano maps and cluster heat maps of differential gene expression.

### Gene Ontology and Kyoto Encyclopedia of Genes and Genomes Enrichment Analysis

Gene Ontology (GO) (go_c, go_f, go_*P*) and Kyoto Encyclopedia of Genes and Genomes (KEGG)_pathway annotations were used, and followed by phyper function in R software to perform enrichment analysis, to calculate the *p*-value, and to perform FDR correction on *p*-value to obtain the Q-values (Wang et al., 2021). Q-values ≤ 0.05 were considered significantly enriched.

### Protein–Protein Interaction Network Construction and Module Analysis

We use the online database STRING to construct the Protein–protein interaction (PPI) network of DEGs, followed by the MCODE plug-in of Cytoscape software to perform module analysis on the constructed PPI network (node score cut-off = 0.2; max. depth = 100; k-core = 10) (Feng et al., 2019). The CytoHubba plug-in was used for hub gene analysis and the Maximal Clique Centrality (MCC) was used for gene sequencing.

### Hub Gene Selection and Analysis

The online tool Kaplan-Meier plotter was used to analyze the correlation between DEGs and the prognosis of ovarian cancer (Shen et al., 2019). The figure showed the log rank *p*-values and hazard ratio (HR) values. To verify the expression of the selected DEGs in ovarian cancer, we used the online website GEPIA to analyze gene expression, which contains the RNA sequencing expression data of thousands of samples of the GTEx and TCGA datasets (Tang et al., 2017). The GeneMANIA online website tool was used for additional protein interaction analysis of the screened genes, and the BiNGO plug-in of Cytoscape was used to analyze the biological process of the screened genes (Maere et al., 2005; Montojo et al., 2010).

### RNA Extraction and cDNA Preparation

RNA extraction was performed in a pre-cooled centrifuge with freshly prepared solutions (chloroform, isopropanol, absolute ethanol). The TransZol Up kit (TransGen Biotech) was used to extract total RNA, according to the manufacturer’s instructions with minor modifications (Meng et al., 2019b). Briefly, the RNA was obtained after washing and centrifugation with 75% absolute ethanol (DEPC water configuration) twice, and then the sample was left to stand at room temperature. After brief drying, 20 μL of RNase-free water was added to resuspend the RNA pellet, and the OD value was assessed to determine the RNA concentration. The A260/280 value was between 1.8 and 2.0, indicating pure RNA. A TransScript One-step gDNA Removal and cDNA Synthesis SuperMix Kit (TransGen Biotech) was used for reverse transcription and cDNA synthesis. Finally, the reverse-transcribed cDNA was stored at -20°C for later use.

### Real-Time Quantitative Polymerase Chain Reaction

Real-time quantitative polymerase chain reaction (RT-qPCR) was used to detect the expression of WT1 genes in different ovarian cancer cell lines. The TB Green™ Premix Ex Taq™ GC kit (TaKaRa) was used for RT-qPCR, and the specific primers used are shown in Table 1. The qPCR reaction program was: 95°C, 30 s; 95°C, 5 s; 57°C, 30 s; 72°C, 30 s; for 40 cycles. The dissolution curve started at 60°C, with temperature increments of 0.5°C every 5 s to 95°C.

**Table 1:** Sequences for gene primers.

Gene Symbol	RT Forward Primer (5′- 3′)	RT Reverse Primer (5′- 3′)
AKT1	AGC​GAC​GTG​GCT​ATT​GTG​AAG	GCC​ATC​ATT​CTT​GAG​GAG​GAA​GT
IRS1	ACA​AAC​GCT​TCT​TCG​TAC​TGC	AGT​CAG​CCC​GCT​TGT​TGA​TG
PCK2	GCC​ATC​ATG​CCG​TAG​CAT​C	AGC​CTC​AGT​TCC​ATC​ACA​GAT
PRKAA2	GTG​AAG​ATC​GGA​CAC​TAC​GTG	CTG​CCA​CTT​TAT​GGC​CTG​TTA
CAT	TGG​AGC​TGG​TAA​CCC​AGT​AGG	CCT​TTG​CCT​TGG​AGT​ATT​TGG​TA
MAPK11	AAG​CAC​GAG​AAC​GTC​ATC​GG	TCA​CCA​AGT​ACA​CTT​CGC​TGA
MYC	GGC​TCC​TGG​CAA​AAG​GTC​A	CTG​CGT​AGT​TGT​GCT​GAT​GT
CDH1	CGA​GAG​CTA​CAC​GTT​CAC​GG	GGG​TGT​CGA​GGG​AAA​AAT​AGG
YAP1	TAG​CCC​TGC​GTA​GCC​AGT​TA	TCA​TGC​TTA​GTC​CAC​TGT​CTG​T
SERPINA	ATG​CTG​CCC​AGA​AGA​CAG​ATA	CTG​AAG​GCG​AAC​TCA​GCC​A
IGFBP1	TTG​GGA​CGC​CAT​CAG​TAC​CTA	TTG​GCT​AAA​CTC​TCT​ACG​ACT​CT
FBN1	TTT​AGC​GTC​CTA​CAC​GAG​CC	CCA​TCC​AGG​GCA​ACA​GTA​AGC
ACTB	CTG​GAA​CGG​TGA​AGG​TGA​CA	AAG​GGA​CTT​CCT​GTA​ACA​ACG​CA
GADPH	AAG​GTG​AAG​GTC​GGA​GTC​AAC	GAA​GGG​GTC​ATT​GAT​GGC​AAC

### Western Blotting Analyses

Western blotting was performed as previously described with minor modifications (Meng et al., 2019a). Briefly, the cells were washed twice with pre-cooled PBS, and RIPA buffer containing protease inhibitors was added to cells, which were lysed on ice for 20 min. The protein lysate was centrifuged at 4°C at 10,000 rpm for 5 min, and the supernatant was added to 5×SDS loading buffer at 100°C for 10 min. The denatured proteins were subjected to SDS-PAGE electrophoresis and were transferred to PVDF membranes (Millipore). After blocking with a blocking solution (Beyotime) for 20 min, the membrane was incubated in the primary antibody at 4°C overnight. The primary antibodies used were rabbit anti-WT1 (1:1,000, Cell Signaling Technology, Inc.), mouse anti-GAPDH, and mouse anti-ACTB (1:1,000, Abclonal). After washing, the Abclonal secondary antibody was incubated at 1:1,000 for 1 h. Finally, the ECL Luminescent Solution (Millipore) was used to visualize blots and for imaging of protein bands.

### Infection of WT1-Expressing Adenoviruses into Ovarian Cancer Cell Line SKOV3

WT1-expressing adenoviruses infected SKOV3, and the cells were cultured as previously described with minor modifications (Meng et al., 2019a; Meng et al., 2019b). Briefly, WT1-expressing adenoviruses infection was performed when the cells reached 50% confluence, and the infected cells were incubated for 24 h. The negative control or adenoviruses containing WT1 infected ovarian cancer cells as recommended by the manufacturer. The adenoviruses (10^10^ PFU/ml) were synthesized by Hanbio Biotechnology.

### Immunofluorescence

Immunofluorescence was performed as previously described with minor modifications (Meng et al., 2019a). Briefly, the cells were fixed with 4% Paraformaldehyde Fix Solution (Beyotime) for 20 min, and then permeabilized in Immunostaining Permeabilization Buffer with Triton X-100 (Beyotime) for 10 min. After blocking with a blocking solution (Beyotime) for 20 min, the cells were incubated in the primary antibody at 4°C overnight. The primary antibodies used were rabbit anti-WT1 (1:200, Cell Signaling Technology, Inc.). The cells were washed with PBS and incubated with the Abclonal secondary antibody was incubated at 1:500 for 1 h. After washing, DAPI staining solution (Beyotime) was incubated for 3 min. After washing, the cells were observed using a fluorescence microscope.

### Proliferation Experiment

The cells in the logarithmic growth phase were seeded into a 96-well plate at 2×10^3^/well. After 24 h of culture, transfection was carried out according to the above steps. At 24 h and 48 h of transfection, add CCK solution (Hanbio Biotechnology) to 10 μL/100 μL culture medium, and incubate in an incubator for 2 h. After that, the absorbance at 450 nm was measured with a microplate reader.

### Transwell Assay

#### Cell Migration Assay

The cells that have been transfected for 24 h were inoculated into the upper chamber of the transwell at a rate of 5×10^4^/well. After the cells adhered to the wall, they were replaced with serum-free culture medium and resuspended, and 10% FBS culture medium was added to the lower chamber. Cells were fixed with 4% paraformaldehyde at 24 h or 48 h of culture, and then stained with 0.1% crystal violet solution, photographed under a microscope, and analyzed with ImageJ software.

#### Cell Invasion Assay

Before inoculating the cells, spread 60 μL of Matrigel (corning) diluted with 6-fold serum-free medium in the upper chamber of the transwell, and place it in a cell culture incubator for 1 h to solidify. The remaining steps are the same as the migration experiment.

### Statistical Analysis

For sequencing data, Eseq2 method was used to correct for DEGs, Q-values <0.05 were considered significantly difference.

For experimental data, GraphPad Prism software was used to statistical analyses. *p* < 0.05 indicated a significant difference, *p* < 0.01 represented a very significant difference. All data were displayed as mean ± standard deviation. The *t*-test and one-way ANOVA analysis were performed to evaluate statistical significance. In evaluating multiple comparisons, Dunnett’s multiple comparisons test was used to improve the accuracy.

## Results

### Wilms tumor gene1 Was Differentially Expressed in Ovarian Cancer and Was Related to Prognosis

**FIGURE 1:**
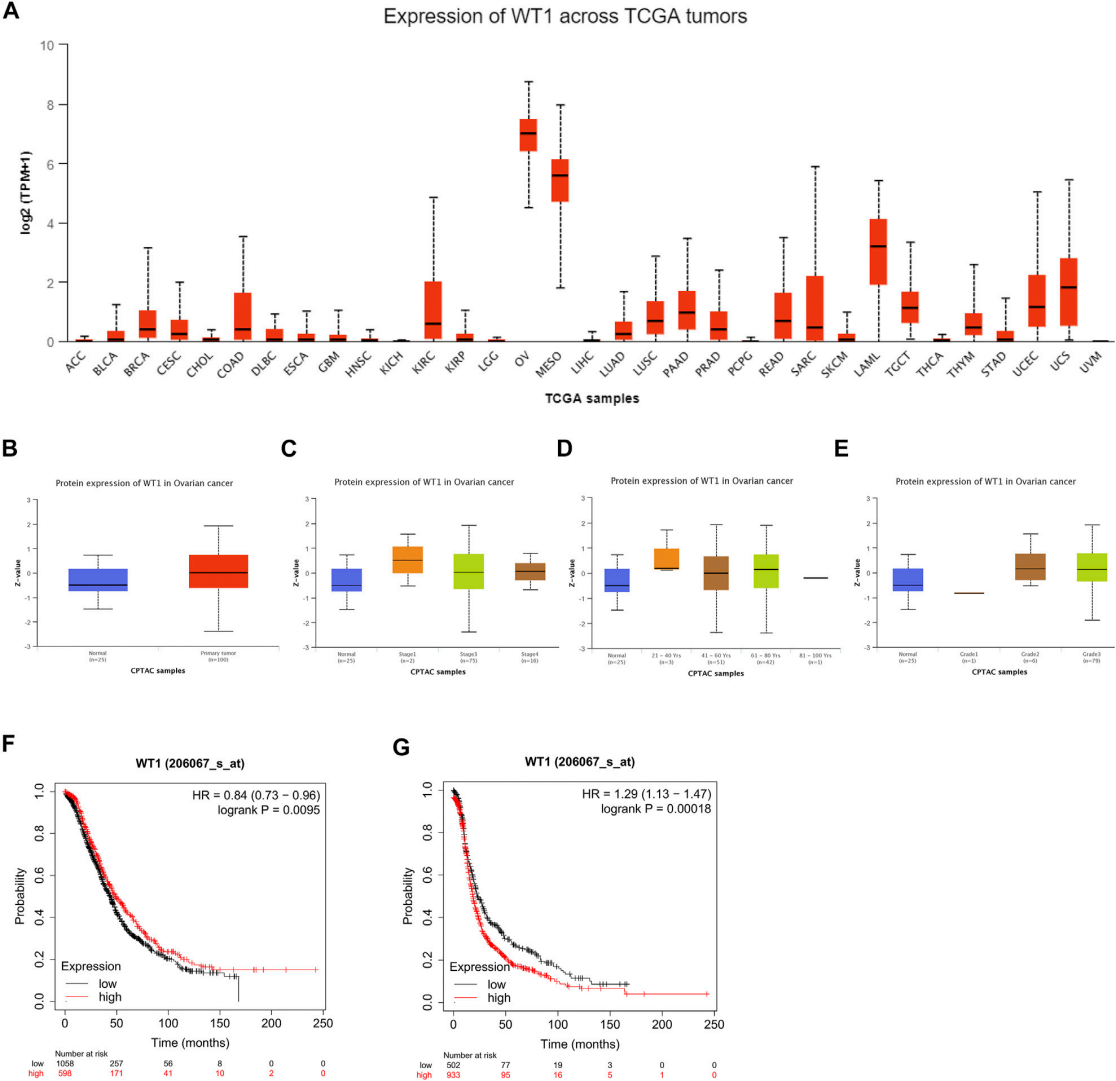
Bioinformatics analysis of the clinical significance of WT1 in ovarian cancer **(A)** The expression level of WT1 mRNA in different cancers was analyzed through the UALCAN website. The expression of WT1 protein in ovarian cancer tissues and normal ovarian tissues **(B)**, ovarian cancers stratified by different stages **(C)**, age **(D)**, and grades **(E)** were analyzed through the UALCAN website. Kaplan-Meier curves showing the correlation between the expression of WT1 and overall survival **(F)** and progression-free survival **(G)** of patients with ovarian cancer.

In order to explore the potential significance of WT1 in ovarian cancer, we used the online website UALCAN to analyze the pan-cancer expression of WT1 and its expression in ovarian cancer of different grades, stages, and ages. The analysis results showed that compared with other cancers, WT1 was highly expressed in ovarian cancer (Figure 1A). WT1 protein expression in ovarian cancer tissues was significantly higher than that in normal tissues (Figure 1B), and was found to be positively correlated with clinical stage, age, and grades of ovarian cancer (Figures 1C–E). We used Kaplan-Meier Plotter online software to analyze the correlation between WT1 and prognosis, and the results indicated that WT1 was correlated with worse survival: log-rank *p* = 0.0095, HR = 0.84 (0.73–0.96) for overall survival (OS) (Figure 1F); log-rank *p* = 0.00018, HR = 1.29 (1.13–1.47) for progression-free survival (PFS) (Figure 1G).

### siRNA Transfection Down-Regulated WT1 Expression in the Ovarian Cancer Cell Line SKOV3

**FIGURE 2:**
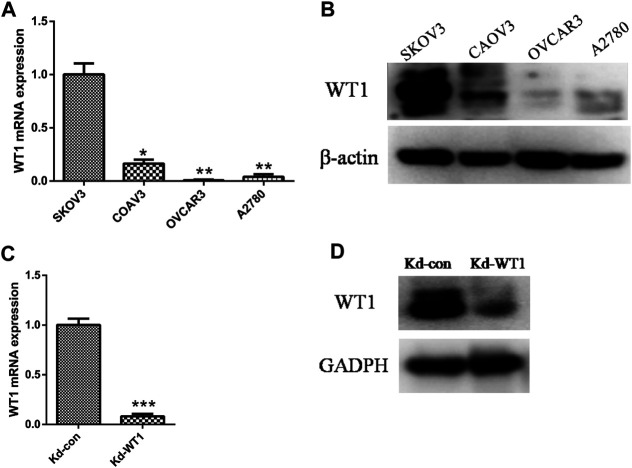
WT1 knockdown in the ovarian cancer cell line SKOV3. **(A,B)** qRT-PCR and Western blotting to detect the expression of WT1 in four ovarian cancer cell lines (SKOV3, Coav-3, OVCAR3, A2780), β-actin acts as control. **(C, D)** qRT-PCR and western blotting were used to detect the expression of WT1 in the SKOV3 cell line after siRNA transfection, GAPDH used as control. *p < 0.05, **p < 0.01, ***p < 0.001. Kd: knockdown.

The results of RT-qPCR and western blotting showed that compared with the other three ovarian cancer cell lines Coav-3, OVCAR3, and A2780, the expression of WT1 in SKOV3 was significantly higher (Figures 2A,B). After transfection of siRNA in SKOV3 cells, the expression of WT1 mRNA and protein was significantly reduced (Figures 2C,D).

### Differential Gene Enrichment Analysis

**FIGURE 3:**
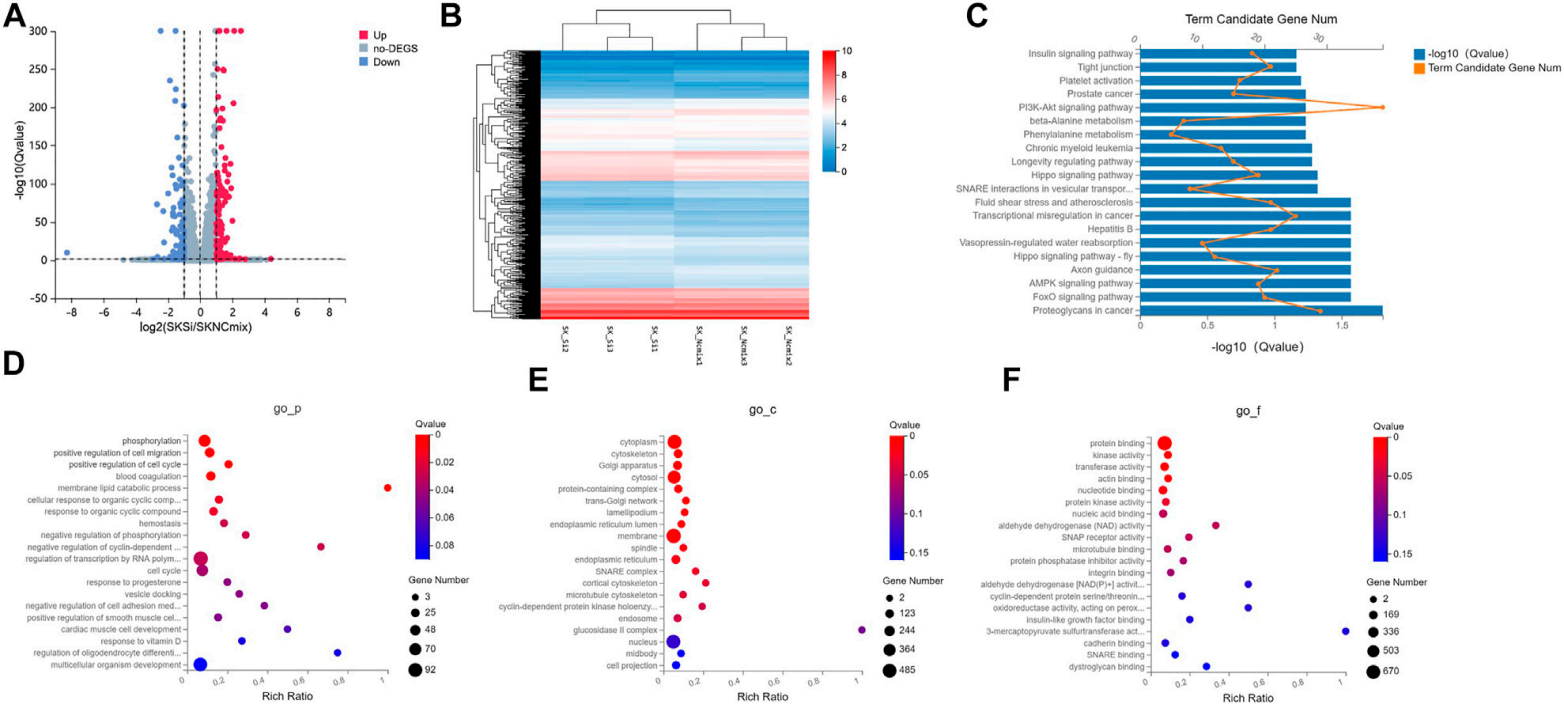
GO and KEGG analysis of DEGs in the ovarian cancer cell line SKOV3. **(A)** Volcano plot of DEGs in the WT1 knockdown group (SKSi) and control group (SKNCmix). **(B)** Heat map of mRNA expression between SKSi and SKNCmix. mRNA expression shown gradually increases from blue to red. **(C)** KEGG analysis of the DEGs in SKOV3. GO analysis of the DEGs in SKOV3 **(D)** Biological processes, **(E)** Cellular components, and **(F)** Molecular functions. KEGG: Kyoto Encyclopedia of Genes and Genomes; GO: Gene ontology; DEGs: differentially expressed genes; go_-_ *P*: biological process in GO analysis; go-c: cellular component in GO analysis; go-f: molecular function in GO analysis.

RNA sequencing detected a total of 1,150 differentially expressed genes (638 up-regulated and 512 down-regulated) compared with the control group. The volcano map showed that there was a large number of differentially expressed genes in the two sets of samples (Figure 3A), and their expression levels were shown in a heat map in Figure 3B. Next, we performed GO and KEGG enrichment analysis on the 1,150 DEGs. The results of KEGG analysis showed that the signal transduction pathways mainly enriched by the DEGs were FoxO, AMPK, and Hippo signaling pathways (Figure 3C). In addition to the signal transduction pathways, the DEGs were also enriched in proteoglycans in cancer, hepatitis B, transcriptional misregulation in cancer, and fluid shear stress and atherosclerosis pathways related to human diseases, and axon guidance related to development. The GO analysis results showed that in addition to processes related to metabolism and stimulus response, the biological processes that were mainly enriched by the DEGs were mainly cell migration, cell cycle, hemostasis, phosphorylation, and cell adhesion (Figure 3D); DEGs were mainly enriched in cellular components such as cytoplasm, cytoskeleton, Golgi apparatus, cytosol and protein-containing complex (Figure 3E); and in terms of molecular function, the DEGs were mainly enriched in protein binding, kinase activity, transferase activity, actin binding, nucleotide binding, and other molecular functions (Figure 3F).

### Analysis of Enriched Genes in the Kyoto Encyclopedia of Genes and Genomes Pathway

**FIGURE 4:**
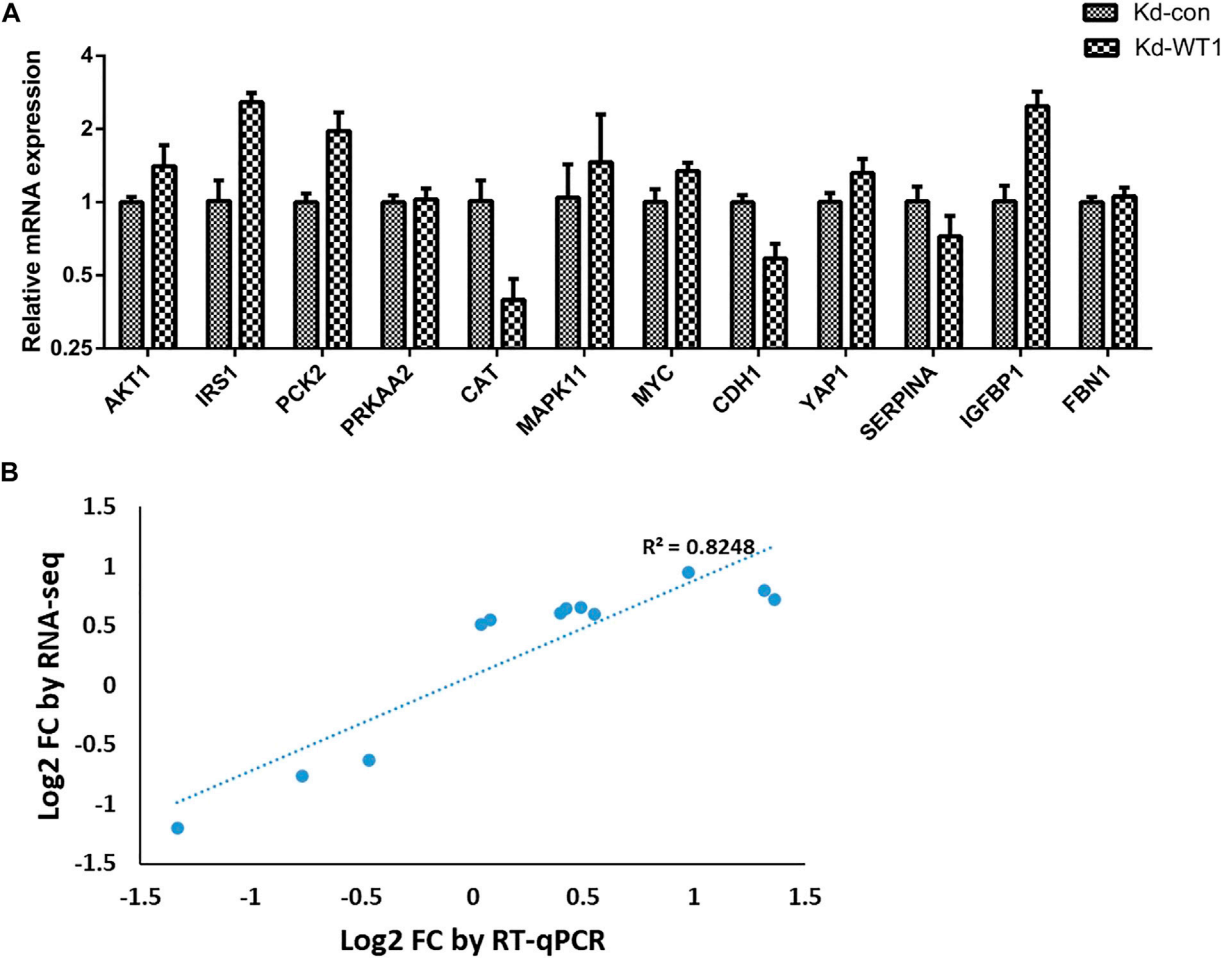
RT-qPCR verification of RNA sequencing data. **(A)** Differential expression levels of 12 screened mRNA detected using RT-qPCR. **(B)** Scatter plots verifying the consistency of the two platforms. GAPDH used as control. Kd: knockdown; con: control; RT-qPCR: reverse transcription quantitative polymerase chain reaction.

We used DEGs that were significantly enriched in the FoxO, AMPK, and Hippo signaling pathways to construct PPI networks through the STRING website, and then we used the CytoHubba plug-in of Cytoscape software to perform hub gene analysis. The hub gene analysis results are shown in Table 2. We selected 9 genes for RT-qPCR verification and the RT-qPCR results are shown in Figure 4A. The pathway diagrams of DEGs enrichment are shown in the Supplementary figures. The expression trend of the selected mRNAs was highly consistent with the RNA-Seq analysis results (Figure 4B).

**Table 2:** The enriched hub genes in KEGG pathway.

KEGG signaling pathways	FoxO	AMPK	Hippo
Rank top 10	AKT1	AKT1	MYC
	CCND1	IRS1	CDH1
	IRS1	PCK2	CCND1
	CAT	SREBF1	CTGF
	MAPK11	LEPR	TGFB1
	CDKN1A	CCND1	ACTB
	CDKN1B	CCNA2	BMP4
	TGFB1	GYS1	YAP1
	SGK1	ELAVL1	BMP2
	CDKN2D	PRKAA2	WNT10A

### Differential Gene Protein-Protein Interaction Network Construction and Core Gene Analysis

**FIGURE 5:**
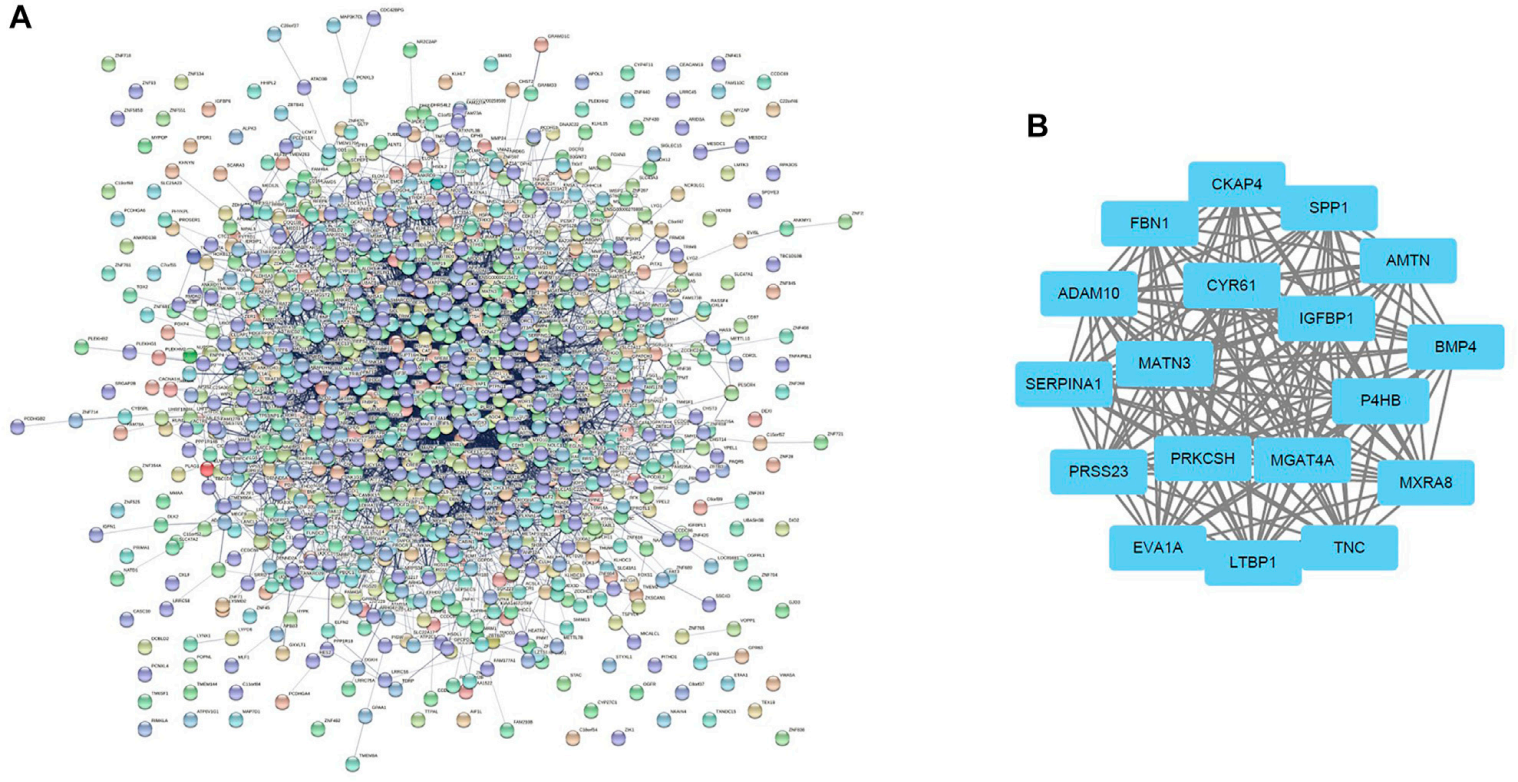
Differential gene expression PPI network construction and module analysis. **(A)** The PPI network of DEGs constructed using the STRING online database. There were a total of 1,150 DEGs in the PPI network. The nodes represent proteins, and the edges represent protein interactions. **(B)** Module analysis using MCODE of Cytoscape software: node score cut-off = 0.2; max. depth = 100; k-core = 10. DEGs: differentially expressed genes.

**FIGURE 6:**
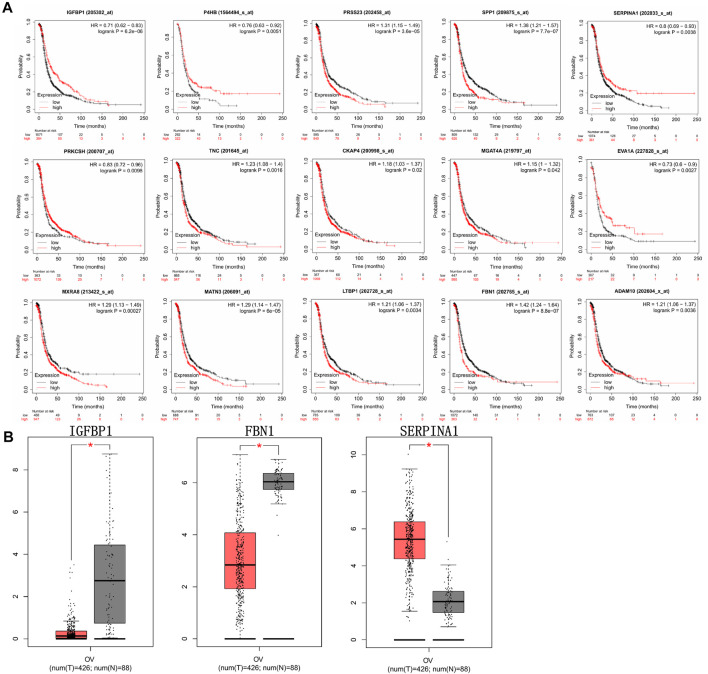
Prognostic information and expression level analysis of hub genes. **(A)** The prognostic information of 18 hub genes was analyzed by Kaplan-Meier plotter online tools, and 16 of the 18 genes were significantly related to the prognosis of ovarian cancer (*p* < 0.05). **(B)** The expression levels of hub genes between ovarian cancer tissues and normal tissues were analyzed through the GEPIA database. Compared with normal tissues, 3 of the 16 genes were significantly differentially expressed in ovarian cancer tissues (*p < 0.05). Red represents tumor tissue; gray represents normal tissue.

**FIGURE 7:**
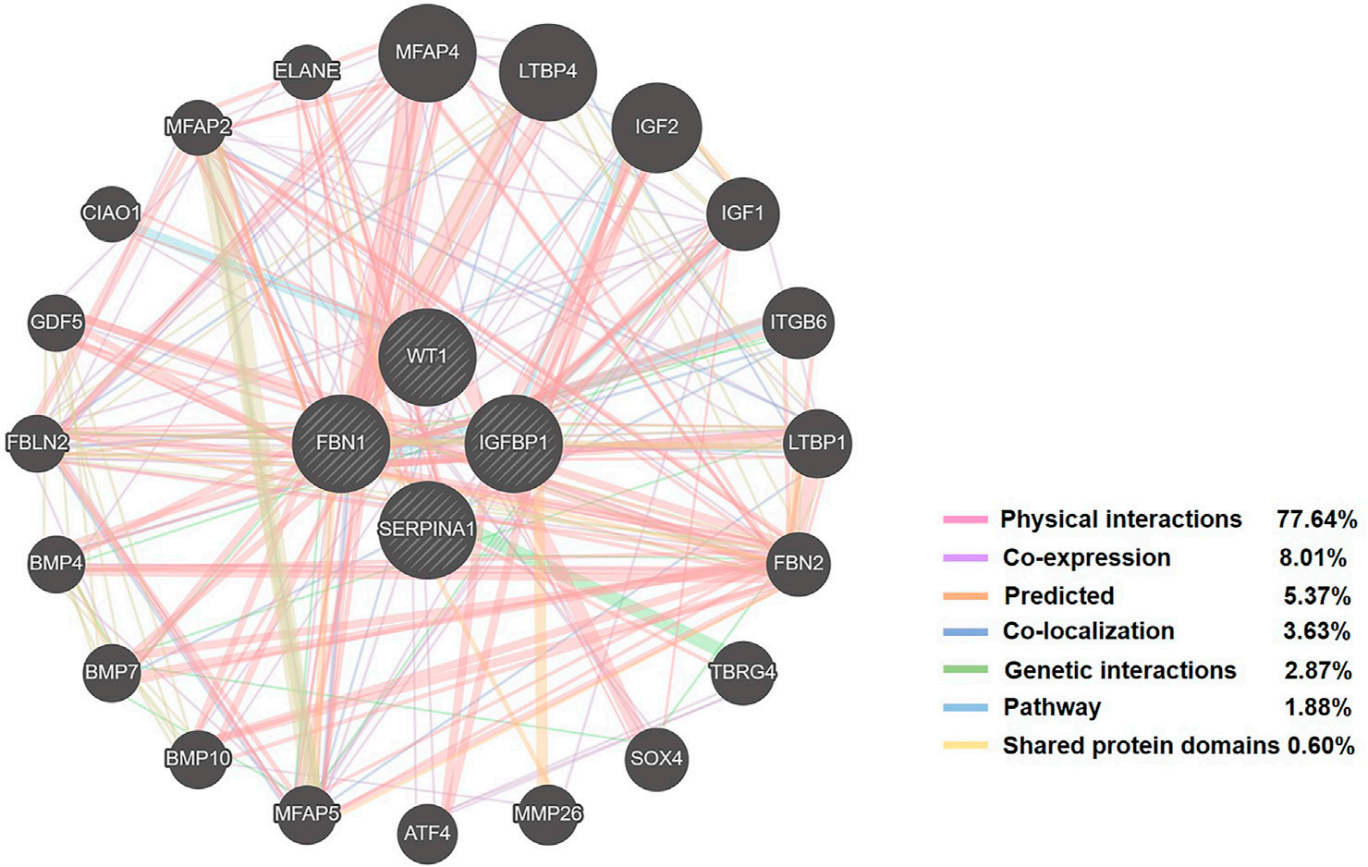
The network of WT1, IGFBP1, FBN1, SERPINA1 and related genes constructed by GeneMANIA.

In total, 1,150 DEGs were imported into the STRING website and PPI network construction was carried out. Twelve of the 1,150 DEGs were not included in the DEGs PPI network (Figure 5A). The MCODE plug-in of Cytoscape software was used for further analysis, and the most important module was selected for subsequent analysis (Figure 5B). Through the Kaplan-Meier plotter online tool, we determined that in addition to BMP4 and AMTN, 16 of the 18 hub genes were related to prognosis (Figure 6A). Next, we used the GEPIA website to compare the expression of 16 genes between tumor tissues and normal tissues. The results showed that 7 genes were differentially expressed between tumor tissues and normal tissues. Among them, the expression of IGFBP1 and FBN1 genes increased significantly after WT1 interference, and the expression of SERPINA1 decreased significantly following interference with WT1 expression (Figure 6B). The RT-qPCR results of these genes are shown in Figure 4A. Using the GeneMANIA online tool, we observed complex interactions between WT1, IGFBP1, FBN1, SERPINA1, MFAP4, LTBP4, IGF2, IGF1, ITGB6, and other 20 genes (Figure 7). In addition, the analysis of the biological process involving WT1, IGFBP1, FBN1, SERPINA1 enrichment is shown in Supplementary Figure S4. The results included 76 nodes and 114 edges. The main biological processes involved included regulation of biological quality, response to endogenous stimulus, regulation of cell growth, wound healing, response to hormone stimulus, and regulation of cell growth.

### The Effect of Wilms tumor gene1 on the Proliferation, Migration and Invasion of Ovarian Cancer Cell Line SKOV3

**FIGURE 8:**
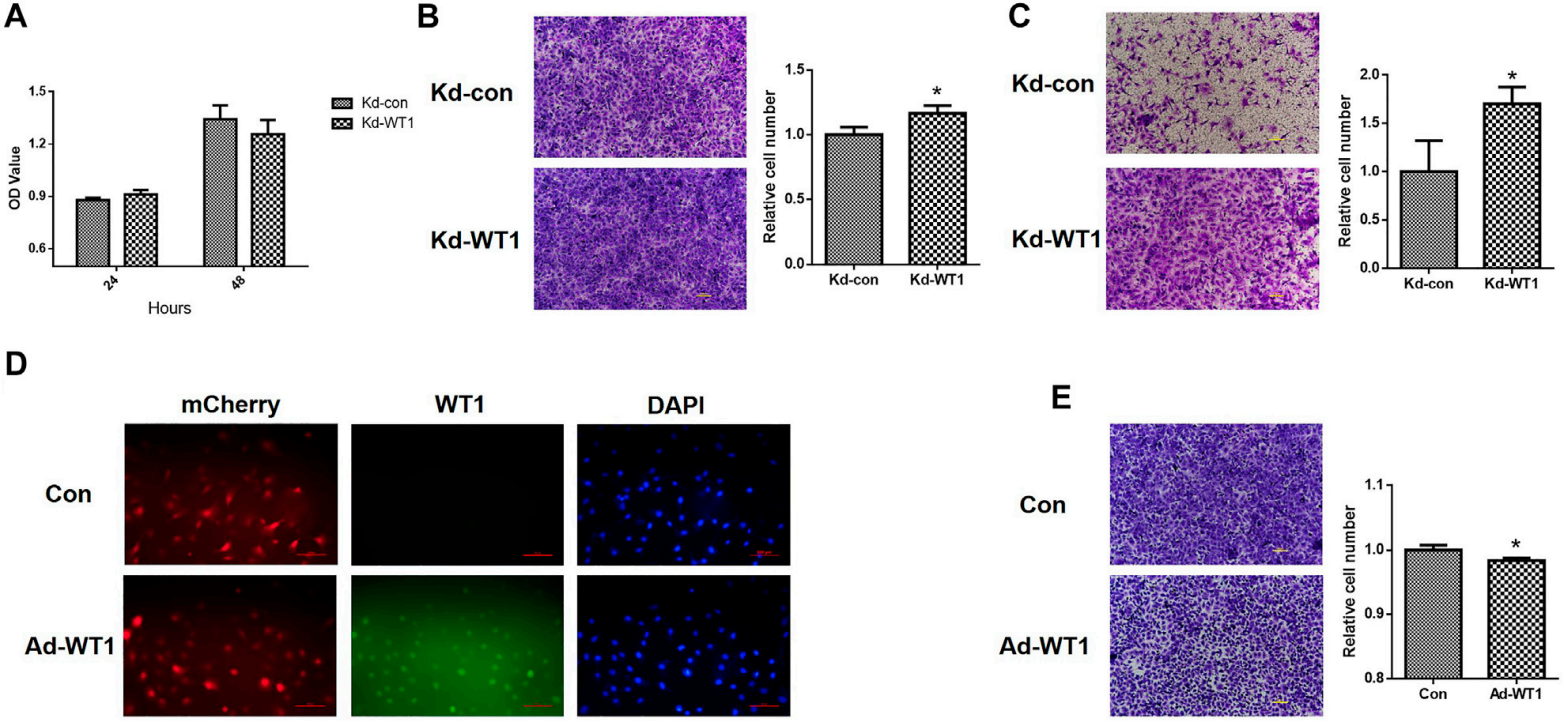
The effect of WT1 on the proliferation, migration and invasion of ovarian cancer cell line SKOV3. **(A)** CCK assay was used to detect the effect of WT1 down-regulation on cell proliferation. **(B)** Transwell cell migration assay to detect the effect of WT1 down-regulation on cell migration ability. **(C)** Transwell cell invasion assay was used to detect the effect of WT1 down-regulation on cell invasion ability. **(D)** Infection efficiency and localization of Con and Ad-WT1 adenoviruses. **(E)** Transwell cell migration assay to detect the effect of WT1 up-regulation on cell migration ability. *p < 0.05. Kd: knockdown, Ad: addition.

CCK and transwell assay were used to verify the effect of WT1 downregulation on the proliferation, migration and invasion of ovarian cancer cell line SKOV3. As shown in Figure 8A, down-regulation of WT1 had no significant effect on the proliferation of SKOV3. Down-regulation of WT1 significantly increased the migration ability of cells (Figure 8B) and the invasion ability of cells was also significantly increased (Figure 8C). Transwell assay was used to further verify the effect of WT1 upregulation on ovarian cancer cell line SKOV3. 24 h after infection of WT1-expressing adenoviruses, the rate of WT1-infection cells was>80% and WT1 protein is mainly located on the nucleus (Figure 8D). As shown in Figure 8E, up-regulation of WT1 significantly inhibited the migration ability of cells.

## Discussion

We used transcriptome sequencing technology to analyze the effects of WT1 on the DEGs in the ovarian cancer cell line SKOV3, and analyzed the signaling pathways, molecular functions, and the associated biological processes, and constructed a PPI network of DEGs and performed module analysis. We analyzed the expression of these genes using the GEPIA website tools, and analyzed the relationship between these genes and the prognosis of ovarian cancer using the Kaplan-Meier plotter online tool, and finally analyzed the interaction between the selected genes and WT1.

Previous studies have shown that compared with normal tissues, WT1 is highly expressed in ovarian cancer (Hylander et al., 2006; Han et al., 2020), and tends to be associated with higher tumor grades and stages, but was not associated with PFS or OS (Hylander et al., 2006). Previous studies have also shown that there are no differences in WT1 staining intensity between normal tissues and ovarian epithelial tumors (Jiang et al., 2014), and the overexpression of WT1 is closely associated with poor prognosis of ovarian cancer (Taube et al., 2016; Carter et al., 2018; Lu et al., 2018; Han et al., 2020). The reason for the above differences may be related to insufficient sample size or heterogeneity of ovarian cancer. In addition, the sensitivity of immunostaining is lower than that of transcriptome sequencing or western blotting. This may also explain why there was no significant difference in WT1 staining intensity. Based on the above, we used bioinformatics methods to provide an in-depth analysis. Our study results indicated that among pan-cancer samples, WT1 showed the highest expression in ovarian cancer. The expression of WT1 in ovarian cancer of different grades and ages was higher than that of normal tissues, and WT1 showed a significant correlation with OS and PFS of ovarian cancer. Although there were differences with the results of previous studies, these data also indicate that WT1 may play an important role in the pathogenesis of ovarian cancer.

In order to further understand the potential mechanisms involved in WT1 regulation of the progression of ovarian cancer, we used the ovarian cancer cell line SKOV3, which highly expresses WT1, as our study focus, and used transcriptome sequencing technology to identify the DEGs influenced by the down-regulation of WT1. We identified a total of 638 up-regulated genes and 512 down-regulated genes. Through GO and KEGG enrichment analysis, the biological functions and signal transduction pathways of these DEGs were further explored. The results of KEGG enrichment analysis showed that the signal transduction pathways mainly enriched for these DEGs were the FoxO signaling pathway, AMPK signaling pathway, and Hippo signaling pathway. These signaling pathways all played important roles in regulating the cancer process. Studies have shown that FoxO protein is involved in cell metabolism, differentiation, proliferation, apoptosis, migration and invasion, and other biological processes (Qiang et al., 2015; Ramezani et al., 2019). The role of FoxO transcription factors in cancer are more complex, as these are associated with the inhibition of cell growth and survival of cancer cells, but they can also promote cancer metastasis and induce cancer drug resistance (Hornsveld et al., 2018). CTCF can affect the progression of prostate cancer by regulating the FoxO signaling pathway (Shan et al., 2019). In addition, FoxO protein plays an important role in platinum resistance in ovarian cancer (Shan et al., 2019). AMPK signaling not only plays an important role in maintaining the balance of energy and metabolism, but it can also control the progression of cancer, inhibit proliferation, migration, and invasion of ovarian cancer cells, and induce cell apoptosis (Liu et al., 2018; Lee et al., 2019; Hsu et al., 2021). Studies have also shown that the Hippo signaling pathway plays an important role in the process of ovarian cancer. The overexpression of YAP1 can promote proliferation, migration, and invasion of SKOV3 and A2780 cell lines, and inhibit their apoptosis (Lin et al., 2020), while inhibiting YAP, a co-activator of the Hippo signaling pathway, can inhibit the drug resistance of ovarian cancer (Muñoz-Galván et al., 2020). In this study, the down-regulation of WT1 increased YAP1 mRNA expression, which may promote the migration and invasion of the SKOV3 ovarian cancer cell line.

There are also complex interactions between WT1 and the FoxO, AMPK and Hippo signaling pathways and hub genes in other cancers. Studies in non-small cell lung cancer (NSCLC) have shown that WT1-interacting protein inhibits cell proliferation and tumorigenicity through the AKT/FoxO1 axis. This protein will damage the phosphorylation and activation of AKT, leading to increased expression and transcriptional activity of FoxO1, inhibiting the expression of cyclin D1, and leading to cell cycle arrest (Wu et al., 2019). AKT1 and CCND1 are two important regulatory proteins in the AMPK signaling pathway. In the human NSCLC cell line A549, WT1 is the transcription factor regulating AKT-1 expression, and its down-regulation can inhibit the expression of AKT1 (Wang et al., 2013). In leukemic cells, the down-regulation of WT1 will cause the down-regulation of CCND1 and MYC, thereby weakening its proliferation and cloning ability (Li et al., 2014). E-cadherin (CDH1) plays an important regulatory role in cell proliferation, cell adhesion, cell polarity and epithelial-mesenchymal transition and other physiological processes, and its disorder may promote tumor proliferation, invasion, migration and transfer (Shenoy, 2019). In the Hippo signaling pathway, WT1 promotes the invasion of NSCLC by inhibiting the expression of CDH1 (Wu et al., 2013). In ovarian granulosa cells, mutations in WT1 can inhibit the expression of CDH1, which may affect the differentiation of granulosa cells and their interaction with oocytes (Wang et al., 2015). In this study, the expression of CDH1 was significantly reduced after down-regulation of WT1, which indicates that in SKOV3 ovarian cancer cells, down-regulation of WT1 to promote its migration and invasion may be partly by inhibiting the expression of CDH1. Studies have also shown that WT1, IGFBP1, and SERPINA1 may be the prognostic biomarkers of metastatic pancreatic cancer, and are of great significance for treatment (Thakur et al., 2008). In liver cancer cells, WT1 can affect the expression of IGFBP1, which may affect the progression and cell survival (Perugorria et al., 2009). The above studies support our findings to a certain extent, indicating that WT1 may play an important role in ovarian cancer through these three signaling pathways and related hub genes.

Tumor migration and invasion are two important hallmarks that influence the treatment and prognosis of ovarian cancer. A better understanding of cancer migration and invasion mechanisms will help to achieve earlier diagnosis and treatment of cancer. In this study, following WT1 gene interference in the ovarian cancer cell line SKOV3, the DEGs were mainly enriched in the FoxO, AMPK, and Hippo signaling pathways. These pathways have been reported to participate in the migration and invasion of cancer cells. Of these, the FoxO signaling pathway is closely related to PI3K/AKT signaling. Studies have shown that FoxO signaling is involved in mediating the proliferation, survival and migration of hepatocellular carcinoma (Li et al., 2021). Similarly, in the study of hepatocellular carcinoma, glycochenodeoxycholate induces AMPK/mTOR-dependent autophagy activation, which in turn promotes cell invasion and migration (Gao et al., 2019). In ovarian cancer cells, researchers found that AMPK/mTOR signaling participates in the regulation of ovarian cancer cell migration and invasion by inducing apoptosis (Lee et al., 2019). The Hippo pathway plays an important role in the migration and invasion of cancer cells. Studies have shown that Hippo-Yap signaling in prostate cancer cells is involved in the regulation of cell migration and invasion (Lai et al., 2019). Yes-association protein (YAP) is a core component of the Hippo pathway. The abnormally high expression of YAP/TAZ is associated with tumor occurrence and progression. Studies have shown that YAP1 knockdown in gastric cancer inhibits cell proliferation and reduces cell invasion and migration (Han, 2019). Our study findings showed that WT1 may not only participate in the regulation of the migration and invasion of ovarian cancer cells through the ERK1/2 pathway, but may also participate in the migration and invasion of ovarian cancer cells by regulating these three signal pathways.

In addition, the core genes IGFBP1, FBN1, and SERPINA1 selected in this study have also been reported to be involved in the regulation of cancer cell migration and invasion. IGFBP1 is an important secretory protein of cells, involved in a variety of biological processes such as cell proliferation, apoptosis, migration, invasion, and adhesion, and has been reported to play an important role in the pathophysiological process of many tumors (Lin et al., 2021). Previous studies have shown that IGFBP-1 in gastric cancer cells can influence cell migration (Luo et al., 2017). In ovarian cancer, the expression of IGFBP-1 is significantly correlated with ovarian risk (Terry et al., 2009). The Fibrillin 1 (FBN1) gene is responsible for encoding a protein called fibrillin 1, which can form filaments, microfibrils, or elastic fibers (Faivre et al., 2003; Davis et al., 2014). Previous studies in gastric cancer cells have shown that miR-133b can inhibit cell proliferation, migration, and invasion by increasing the expression of FBN1 (Yang et al., 2017). Interfering with the expression of FBN1 in osteosarcoma cells inhibits cell migration and invasion (Liu et al., 2020). Serpin peptidase inhibitor clade A member 1 (SERPINA1), a protease inhibitor plays an important role in a variety of cancers. Previous studies have shown that the expression of SERPINA1 is associated with poor prognosis in patients with lung, colon, and skin cancer (Farshchian et al., 2011; Chan et al., 2015; Kwon et al., 2015; Ercetin et al., 2019). In addition, SERPINA1 has also been reported to be involved in the regulation of ovarian cancer cell migration and invasion (Normandin et al., 2010). Therefore, IGFBP1, FBN1, SERPINA1 not only have complex interactions with WT1, but also may jointly regulate the migration and invasion of ovarian cancer cells.

Previous studies have shown that WT1 can be used as an important marker for the diagnosis and prognosis of ovarian cancer (Basak et al.; Taube et al., 2016; Gaaib, 2018; Sallum et al., 2018; Mondal et al., 2021). Our research has identified related genes and signaling pathways in ovarian cancer that are affected by WT1, and identified genes that are associated with WT1 and affect the prognosis of ovarian cancer. The combined detection of these genes may help improve the accuracy and consistency of ovarian cancer diagnosis. In addition, vaccines or drugs currently developed targeting WT1 have been used in cancer treatment, and some of them may be combined with conventional chemotherapy or other drugs to treat cancer (Izumoto et al., 2008; Hashimoto et al., 2015; Ogasawara et al., 2019). However, long-term administration to a target site may lead to acquired drug resistance, causing many cancer patients to experience recurrence and progression (Engelman and Jänne, 2008). Our study has identified potential therapeutic targets and signaling pathways associated with WT1 expression, which may lead to the development of targeted drugs for the treatment of ovarian cancer to increase the efficacy of combination treatment.

In conclusion, our research not only helps to further enrich the possible involvement of WT1 in the occurrence and development of ovarian cancer, especially the regulatory mechanisms of migration and invasion, but also provides a theoretical rational for establishing WT1 as a suitable target for treatment and intervention of advanced ovarian cancer.

## Data Availability

The datasets presented in this study can be found in online repositories. The data presented in the study are deposited in the NCBI SRA repository, accession number : SRP337713.
