# Supplementary figures and images for: Application of Bioinformatics Analysis to Identify Important Pathways and Hub Genes in Ovarian Cancer Affected by WT1
Source: Front Bioeng Biotechnol. 2021 Oct 6;9:741051. doi: 10.3389/fbioe.2021.741051 (PMC8526536; doi:10.3389/fbioe.2021.741051)

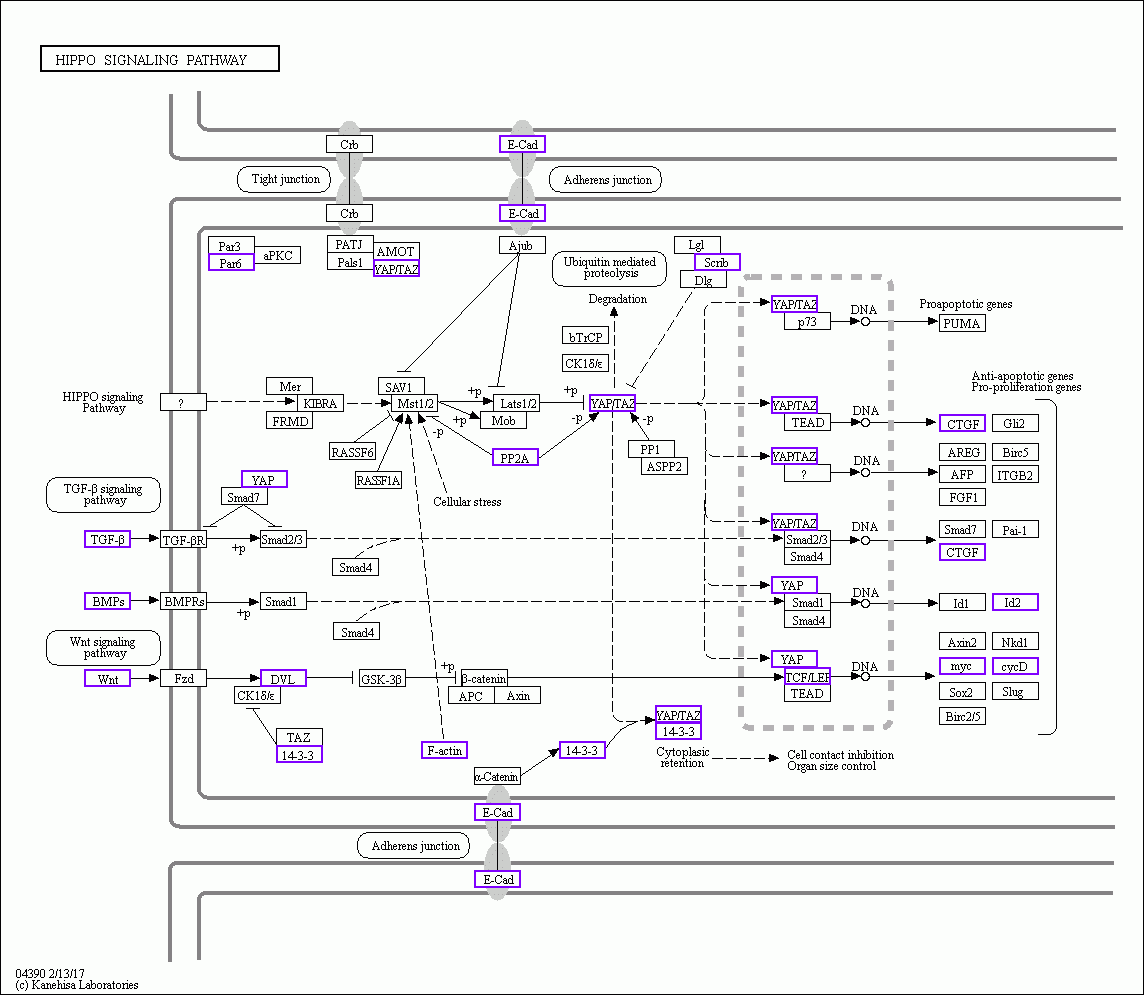

Supplement: Supplementary file 1 [file Image3.TIF]

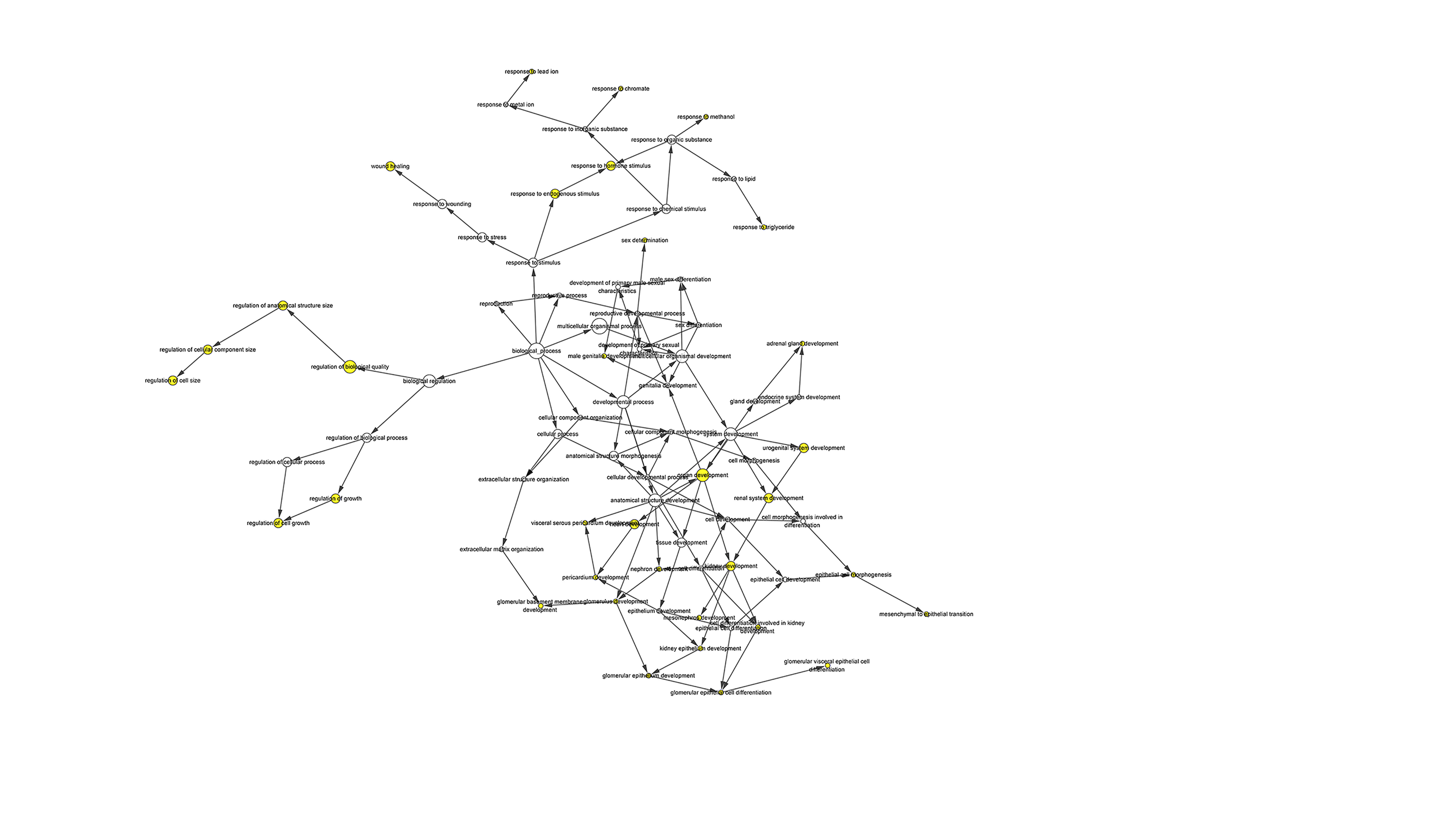

Supplement: Supplementary file 2 [file Image4.TIF]

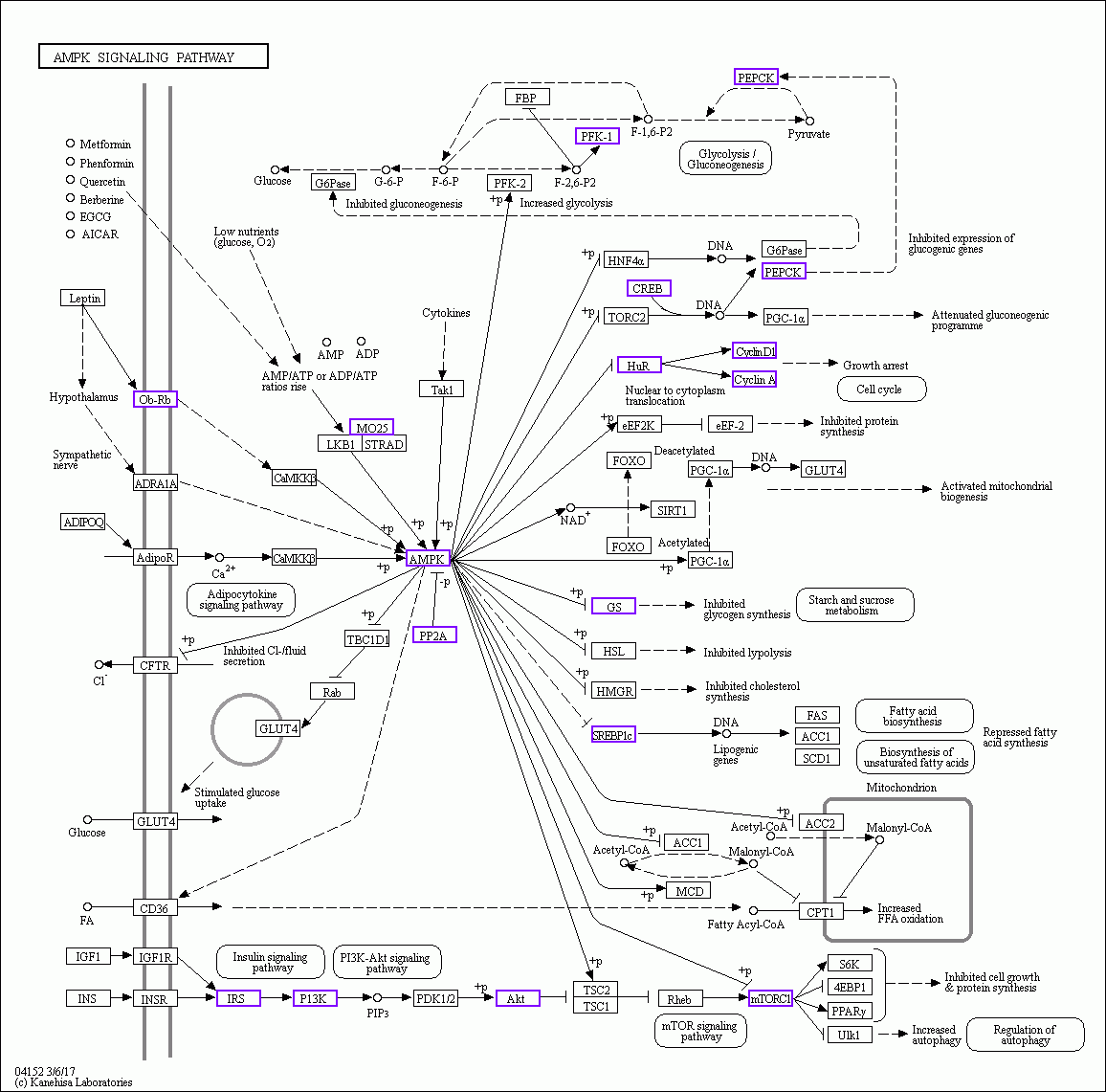

Supplement: Supplementary file 3 [file Image2.TIF]

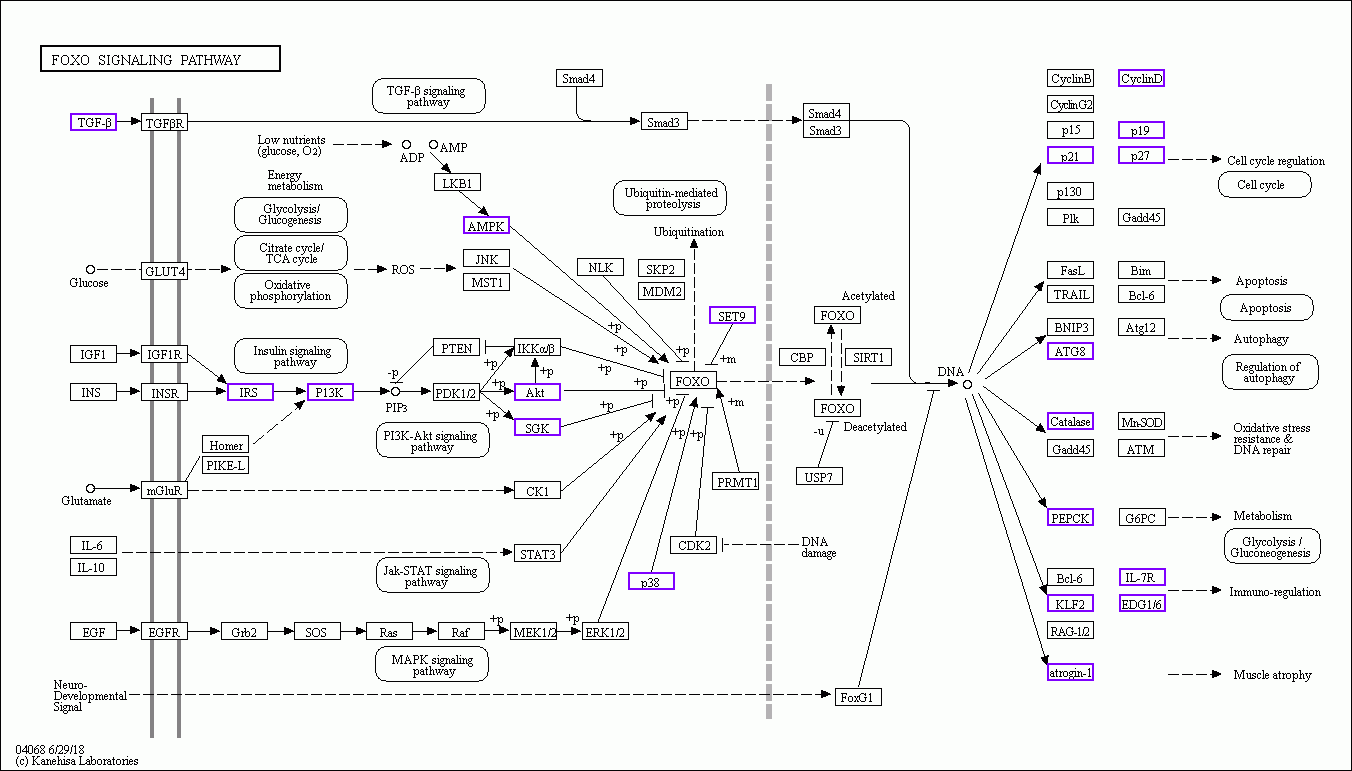

Supplement: Supplementary file 4 [file Image1.TIF]
